# Model of the Distribution of Diastolic Left Ventricular Posterior Wall Thickness in Healthy Adults and Its Impact on the Behavior of a String of Virtual Cardiomyocytes

**DOI:** 10.1007/s12265-014-9558-4

**Published:** 2014-03-28

**Authors:** Kamil Fijorek, Felix C. Tanner, Barbara E. Stähli, Grzegorz Gielerak, Pawel Krzesinski, Beata Uzieblo-Zyczkowska, Pawel Smurzynski, Adam Stanczyk, Katarzyna Stolarz-Skrzypek, Kalina Kawecka-Jaszcz, Marek Jastrzebski, Mateusz Podolec, Grzegorz Kopec, Barbara Stanula, Maryla Kocowska, Zofia Tylutki, Sebastian Polak

**Affiliations:** 1 Department of Statistics, Cracow University of Economics, Krakow, Poland; 2 Cardiology, Cardiovascular Center, University Hospital Zurich, Zurich, Switzerland; 3 Department of Cardiology and Internal Medicine, Military Institute of Medicine, Warsaw, Poland; 4 First Department of Cardiology, Interventional Electrocardiology and Hypertension, Jagiellonian University Medical College, Krakow, Poland; 5 First Department of Cardiology, Interventional Electrocardiology and Hypertension, University Hospital, Krakow, Krakow, Poland; 6 Department of Coronary Artery Disease, Jagiellonian University Medical College at the John Paul II Hospital, Krakow, Poland; 7 Department of Cardiac and Vascular Diseases, Jagiellonian University Medical College and Centre for Rare Cardiovascular Diseases at the John Paul II Hospital, Krakow, Poland; 8 Eskulap Medical Center, Tarnow, Poland; 9 University Hospital in Krakow, Krakow, Poland; 10 Unit of Pharmacoepidemiology and Pharmacoeconomics, Faculty of Pharmacy, Jagiellonian University Medical College, Krakow, Poland

**Keywords:** Left ventricle, Echocardiography, Left ventricular posterior wall thickness, Drug safety, Cardiotoxicity

## Abstract

Correlation of the thickness of the left ventricular posterior wall (LVPWd) with various parameters, including age, gender, weight and height, was investigated in this study using regression models. Multicenter derived database comprised over 4,000 healthy individuals. The developed models were further utilized in the in vitro–in vivo (IVIV) translation of the drug cardiac safety data with use of the mathematical model of human cardiomyocytes operating at the virtual healthy population level. LVPWd was assumed to be equivalent to the length of one-dimensional string of virtual cardiomyocyte cells which was presented, as other physiological factors, to be a parameter influencing the simulated pseudo-ECG (pseudoelectrocardiogram), QTcF and ∆QTcF, both native and modified by exemplar drug (disopyramide) after *I*_Kr_ current disruption. Simulation results support positive correlation between the LVPWd and QTcF/∆QTc. Developed models allow more detailed description of the virtual population and thus inter-individual variability influence on the drug cardiac safety.

**Electronic supplementary material:**

The online version of this article (doi:10.1007/s12265-014-9558-4) contains supplementary material, which is available to authorized users.

## Introduction

Echocardiography has become one of the dominant cardiac imaging techniques as it has good temporal and spatial resolution, and it is widely available given its low costs compared to other imaging modalities. This imaging technique is also supported by the recommendations published under the auspices of two major scientific cardiological societies — the American Society of Echocardiography and the European Association of Echocardiography [1]. Echocardiography allows a detailed evaluation of the structure and function of the heart, assessment of the function of the heart valves as well as evaluation of potential congenital and acquired heart diseases [2]. There are many parameters that are routinely assessed by echocardiography, e.g., wall thicknesses, ventricular dimensions, volumes, masses and ventricular function [3, 4]. In particular, measurements of the left ventricular structure and function are widely utilized in, e.g., daily routine as well as intensive and postoperative care.

There has been only a handful of studies investigating the effect of different demographic and physiological parameters on the left ventricular posterior wall thickness (LVPWd) in healthy adults. The fact that they were written in 1990s can potentially leads to concerns regarding their consistency with the best current clinical practice, both in terms of clinical guidelines and accuracy of measurement equipment and measurement technique applied. Sjögren provides a linear regression model correlating the LVPWd with age, separately for males and females. This study, performed in a group of healthy individuals (58 women and 42 men, aged 18–61 years), was based on echocardiographic measurements [5]. According to Sjögren, the mean LVPWd can be approximated using the following two equations: 0.055 × age + 5.95 (equation for males), 0.096 × age + 3.83 (equation for females). The mean free heart wall thickness was investigated by Henry and colleagues [6]. Their study group consisted of 136 healthy subjects (78 men and 58 women, aged 20–97 years), but the sex of the subjects was not included in their regression models. According to Henry, the mean free left ventricular wall thickness can be approximated with similar accuracy using any of the following two equations: 5.56 × body surface area (BSA)^0.5^ + 0.03 × age + 1.1, 1.92 × weight^0.32^ + 0.03 × age + 1.1. However, Henry’s model lacks a clear definition of the area of the ventricular wall used for the measurements, i.e., non-specific free wall thickness is mentioned rather than posterior wall thickness. Kitzman and colleagues investigated the age-, sex- and BSA-related differences in human adult (20–99 years old) hearts based on autopsy specimens [7]. The authors found no significant correlations between any of the predictors and the left wall thickness in a group of 765 healthy adults. In this study, however, the analyzed variable was not exactly the LVPWd but average wall thickness measured at various sites of the left ventricle. There were also LVPWd models developed for pediatric populations, but since they are out of the scope of the current study, we will only mention the Carceller and colleagues study [8]. They developed a model correlating BSA with the LVPWd, based on a group of 69 healthy individuals aged 10–20 years.

Drug-induced cardiotoxicity, with pro-arrhythmic activity as the leading one, remains a clinical problem and novel methods of early assessment of this phenomenon are intensely discussed [9, 10]. Current pre-clinical approaches are based on in vivo animal studies (dog as a leading species) and in vitro studies assessing *I*_Kr_ current inhibition carried out with the use of human ion channels expressed heterologously in various cells [11]. Modifications of these approaches have recently been proposed to account for more currents analysis and more thorough data integration, due to a likely high level of false positive signals [12]. Hence, mathematical models integrating in vitro data are likely to become an important element of the new paradigm [37]. These biophysically detailed mathematical models (BDMM) describe the electrophysiology of human left ventricular cardiomyocytes. All of them are based on the so-called Hodgkin–Huxley paradigm describing how action potential is propagated in excitable cells [13]. In principle, it is a set of differential equations describing ions flow through the ion channels and pumps with relation to the external excitation and cell capacitance. In BDMMs, the heart wall is assumed to be equivalent to the one-dimensional string of virtual cardiomyocyte cells, and the heart wall thickness is assumed to be equivalent to the length of the string of virtual cells [14–16]. To that end, it is of great interest to investigate whether the LVPWd is a relevant element of the BDMM, apart from other parameters describing physiological factors, discussed previously in several studies [17–19]. Utilization of system information (human physiology data) could allow for the intra- and inter-individual variability assessment [38].

This study serves two strongly interconnected goals: (1) to develop a regression model describing the effect of age, sex, and BSA on the distribution of the LVPWd in healthy adults and (2) to transfer these findings on a biophysically detailed mathematical model describing the electrophysiology of the human left ventricular cardiomyocytes.

## Materials and Methods

### Clinical Data Characteristics

Clinical data were collected retrospectively in Switzerland and Poland. The Swiss data collection was carried out at the University Hospital Zurich (UHZ; *N* = 4,472). Echocardiography studies were performed between 1990 and 2011 (95% of data after 2001). Detailed information regarding data collection has been previously published [20]. The Polish data collection was carried out by the Department of Cardiology and Internal Diseases of the Military Institute of Medicine (Warsaw), the First Department of Cardiology, Interventional Electrocardiology and Hypertension, University Hospital (Krakow), the Department of Cardiac and Vascular Diseases at the John Paul II Hospital (Krakow) and the Eskulap Medical Center (Tarnow). Echocardiography studies were performed in 2002 (*N* = 36) and between 2008 and 2013 (*N* = 281).

The inclusion criteria were as follows: (1) LVPWd in a physiological range 6–11 mm (range according to the current clinical recommendations), (2) lack of known hypertension and other cardiovascular diseases, (3) individual age equal or greater than 18 years, and (4) body mass index (BMI) in a physiological range (16–35) [1]. In all cases, subjects provided informed consent.

### Models Development Methodology

The aim of this section is to describe the steps undertaken to create a regression model of the relationship between the LVPWd and age, BSA and sex. BSA was calculated according to the widely accepted approximation BSA ≅ 0.20247 ⋅ Height (m)^0.725^ ⋅ Weight (kg)^0.425^ [21]. As previously noted, according to the medical diagnostic guidelines, the LVPWd in healthy humans takes on values in the interval 6–11 mm. Due to this restriction, we decided to model the distribution of the LVPWd as a continuous limited random variable. The current state of knowledge in the area of modeling this kind of variables suggests that the preferred approach is the so-called 'beta regression'. The following exposition of 'beta regression' follows closely the Cribari-Neto and Zeileis study [22].

The usual practice while performing a regression analysis in which the dependent variable (response variable, *y*) takes on values in the interval (*a*, *b*) (with *a* < *b* known) is to completely ignore this fact and perform an ordinary regression analysis as if the dependent variable assumed values in the real line. This approach, nonetheless, has many limitations. The most important one in the context of this study is that the simulations from a model not respecting the natural limits of the dependent variable may generate values outside the (*a*, *b*) limits. Better yet, although it is still not optimal, in this approach we first use the linear transformation *y* = (*y* − *a*)/(*b* − *a*) (in the case considered in this article *a* = 6 and *b* = 11), after which *y* assumes values from 0 to 1. The next step is to logit-transform the data so that the transformed response assumes values in the real line, and then to apply a standard linear regression analysis. This approach, however, suffers from other limitations. First, regressions involving data from the finite interval are typically heteroscedastic, i.e., they display more variation around the mean, and less variation around the lower and upper limits of the interval. Second, the distribution of limited variable is typically asymmetric, and thus Gaussian-based approximation for estimation, hypothesis testing and simulation can be inaccurate. Ferrari and Cribari-Neto [23] proposed a regression model for continuous variable that assumes values in the standard unit interval. Since the model is based on the assumption that the response is beta-distributed, they called their model the beta regression model. The model is naturally heteroscedastic and easily accommodates asymmetries. A generalization of the beta regression model was proposed by Simas et al. [24]. In this model, the parameter accounting for the precision of the data is not assumed to be constant across observations but it is allowed to vary, leading to the variable dispersion beta regression model (VDBRM). The VDBRM model will be employed in our analysis of the LVPWd.

The VDBRM is based on an alternative parameterization of the beta density in terms of the mean (*μ*) and precision parameter (*ϕ*):

$$ f\left(y;\mu, \phi \right)=\frac{\Gamma \left(\mu \right)}{\Gamma \left(\mu \phi \right)\Gamma \left(\left(1-\mu \right)\phi \right)}{y}^{\mu \phi -1}{\left(1-y\right)}^{\left(1-\mu \right)\phi -1} $$

with *y* ∈ (0, 1), *μ* ∈ (0, 1) and *ϕ* > 0. Let *y*_1_, *y*_2_, …, *y*_*n*_ be a sample of independent data-points such that *y*_*i*_ has *f*(*y*_*i*_; *μ*_*i*_, *ϕ*_*i*_) distribution. The VDBRM is defined as:

$$ \begin{array}{c}{g}_1\left({\mu}_i\right)={\beta}_0+{x}_{1i}{\beta}_1+\cdots +{x}_{ki}{\beta}_k\\ {}{g}_2\left({\phi}_i\right)={\gamma}_0+{z}_{1i}{\gamma}_1+\cdots +{z}_{pi}{\gamma}_p\end{array}, $$

where *g*_1_ and *g*_2_ are logistic and logarithmic link functions, *x* and *z* are independent variables (covariates), *β* and *γ* are unknown regression parameters.

In this study, model parameters were estimated by the method of bias-reduced maximum likelihood as implemented in the *betareg* package in the R system for statistical computing [22]. A model fit was described using the coefficient of pseudo-determination *R* ^2^. Model adequacy was assessed using different types of diagnostic plots: residuals vs. indices of observations, Cook’s distance plot, generalized leverage vs. predicted values, residuals vs. linear predictor, half-normal plot of residuals, predicted vs. observed values.

In search of the most parsimonious model specification we employed the backward stepwise strategy. The starting models included linear effects of quantitative variables, dummy sex variable (1 = male) and pairwise interaction terms. All mentioned terms were included in both mean and precision model equations. The elimination of predictors was guided by the likelihood ratio test. The modeling exercise was separately performed on Polish and UHZ data

In addition to regression modeling, a tool for simulation of random individual LVPWd, given values of independent variables from estimated models is provided. The tool takes form of an Excel spreadsheet (electronic supplement) that uses built-in standard beta cumulative distribution function. The necessary translation between reparameterized and standard beta distribution is described in detail by Ferrari and Cribari-Neto [23].

### Electrocardiogram (ECG) Simulation Methodology

The LVPWd was used as a surrogate of the left heart wall thickness and it was hypothesized that by modifying the length of the string of virtual cells according to the developed LVPWd models one can influence the electrophysiological model outputs and therefore more reliably predict the clinically expected inter-individual variability [25]. Two BDMM’s outputs were simulated and both of them were derived from the simulated pseudo-ECG traces, i.e., QT and QT corrected by the heart rate with the Fridericia equation (QTcF). The computer simulations were designed and carried out using the Cardiac Safety Simulator (CSS platform). It is worth mentioning that every element of the system including physiological parameters and their variability was described in a form of a scientific publications with all necessary algorithms included. For details regarding the CSS and abovementioned publications see certara.com, tox-portal.net and citations enumerated in this section. The ten Tusscher model of the human left ventricular cardiomyocyte built-in to the CSS and utilized for the current study is considered to be one of the state-of-the-art models in its field [26]. The Forward Euler method was employed to integrate model equations. A one-dimensional fiber of cardiomyocytes comprised of 50% endocardial, 30% midmyocardial and 20% epicardial cells was constructed for each simulated virtual individual. All other physiological parameters describing virtual individuals, namely cardiomyocytes morphometric parameters (volume, area, electric capacitance), plasma ions concentration (K^+^, Na^+^, Ca^2+^) and heart rate were specific for healthy individuals, and CSS default parameters for the empirical models describing inter-individual variability in population were utilized (see summary in Table 1). All virtual individual’s physiological parameters were kept constant across simulated scenarios in order to remove unwanted heterogeneity that might otherwise obscure observation of the LVPWd impact on the simulated endpoints. Simulations were carried out for 60 virtual individuals (30 males, 30 females). All of them were wild type ionic channels genetic variant carriers.

**Table 1:** Summary of the physiological parameters used for the simulation study

Parameter	Unit	Mean (SD)
Plasma potassium concentration	mM	4.29 (0.28)
Plasma sodium concentration	mM	139.37 (1.26)
Plasma calcium concentration	mM	2.36 (0.18)
Cardiomyocyte volume	μm^3^	7254.90 (4912.68)
Stimulation period	ms	909.37 (136.20)
Electric capacitance	pF	55.16 (32.91)

All parameters derived randomly from the models (references to the models used for deriving the above listed values can be found in the text [17–19]) describing parameter distribution in the population of healthy individuals (60 virtual individuals)

Three simulation scenarios were evaluated. In the first scenario, the string length was set to a constant value calculated as the average of all available LVPWd data (8.3 mm). In the second scenario, the LVPWd was randomly drawn from the Sjögren model whose shortcomings were described in the Introduction. However, this model is currently available as an option in the CSS platform and for this reason such a scenario has been included. In the third scenario, the LVPWd was randomly drawn from the age–sex LVPWd model developed on Polish data (abbreviated as ASLPM). Among all the models developed in this study, this one seemed to be of the highest practical relevance. Each scenario included two subscenarios – with and without the test drug (disopyramide), which was assumed to inhibit *I*_Kr_ ionic current in the concentration dependent manner. The Hill equation parameters provided as the input data for CSS were 9.64 μM and 1 for the IC_50_ and *N*, respectively, which resulted in 62% of the *I*_Kr_ current inhibition for the tested active concentration (15 μM) [27]. Distinction between drug/no drug scenarios allows us to evaluate the impact of the LVPWd under markedly different, yet clinically important, situations since BDMMs are increasingly used as a tool for early drug safety assessment [28–31, 34, 35].

## Results

### Modeling Results

In Table 2, sample sizes along with basic descriptive characteristics (mean, standard deviation, minimum − maximum) of the collected data are presented.

**Table 2:** Characteristics of the clinical and echocardiographic data used for the modeling purposes

		UHZ data set	Polish data set
Sample size	Males	2,104	189
	Females	2,368	128
Age (years)	Males	42.1 (13.2, 18.0–79.8)	36.6 (13.6; 18–75)
	Females	42.9 (13.1, 18.0–78.4)	42.3 (14.1; 18–71)
BSA (m^2^)	Males	1.95 (0.17, 1.34–2.73)	2.0 (0.16; 1.53–2.44)
	Females	1.69 (0.15, 1.28–2.34)	1.69 (0.13; 1.44–2.04)
BMI (kg/m^2^)	Males	24.7 (3.4, 16.2–35.0)	26.1 (3.4; 18.4–34.3)
	Females	23.3 (3.9, 16.1–34.8)	24.3 (3.9; 17.0–34.4)
LVPWd (mm)	Males	8.76 (1.04, 6.1–10.9)	9.55 (0.97; 7–10.94)
	Females	7.78 (0.98, 6.1–10.9)	8.63 (1.22; 6.1–10.9)

Values presented as mean (standard deviation, minimum – maximum)

**Fig. 1:**
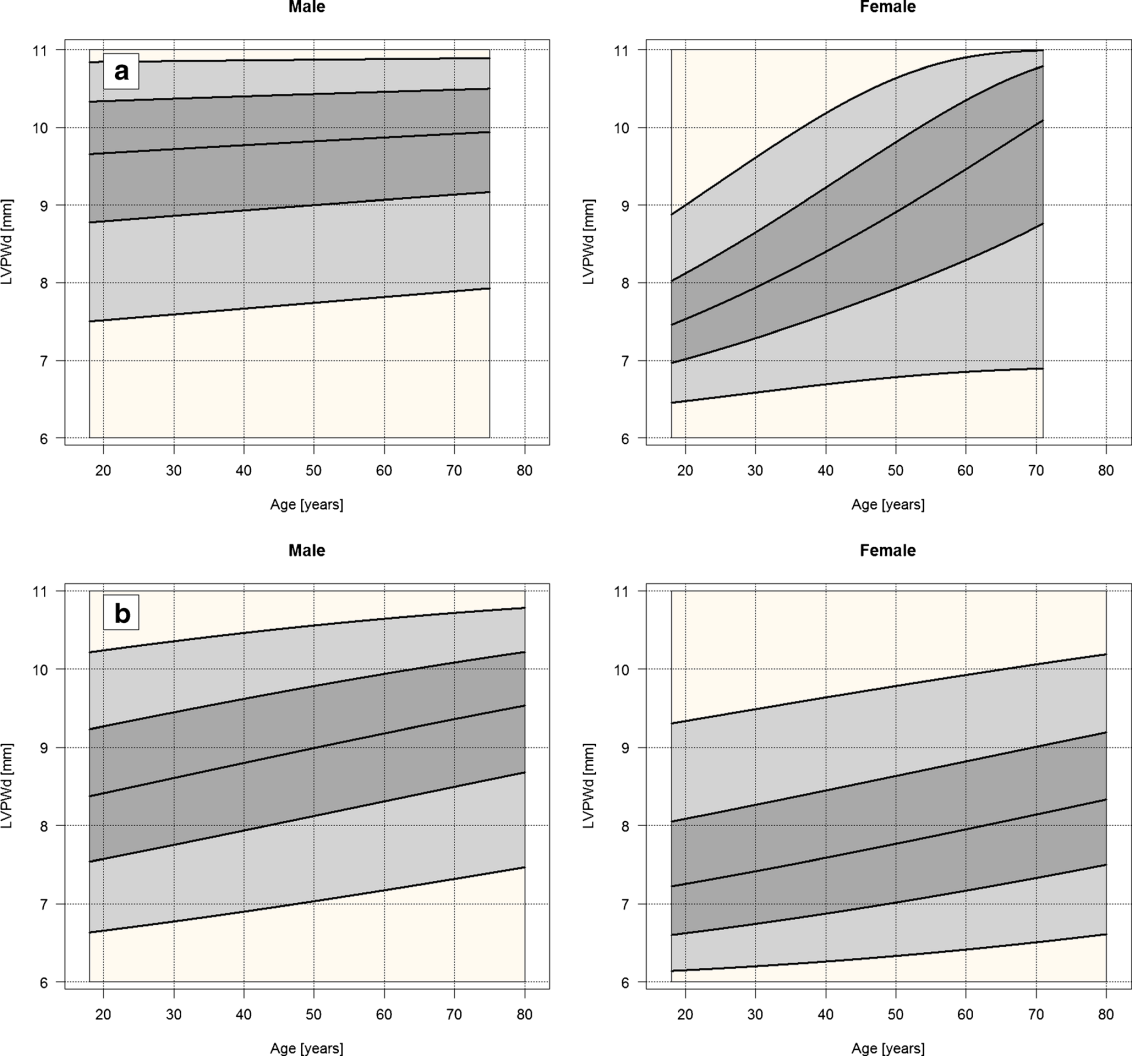
Relationship between LVPWd and age calculated with use of the developed models. **a** Polish data, **b** UHZ data. The *bold curves* from the *bottom* to *top* describe 5th, 25th, 50th, 75th and 95th conditional percentiles of the LVPWd, respectively

Estimation results of the first model are reported in Table 3, separately for Polish and UHZ datasets. In this model, only age and sex were allowed to enter model equations. Model coefficients in the mean submodel are interpretable in terms of the mean of scaled and logit transformed LVPWd, and not in terms of the mean of the original LVPWd. Consequently, we aid our interpretation by plotting model results. Figure 1 shows the estimated relationship between LVPWd and both age and sex, also separately for Polish and UHZ datasets. Since the LVPWd is a limited variable, as mentioned before, the *y*-axis is constrained to the interval from 6 to 11 mm.

**Table 3:** Parameter estimates, 95% confidence intervals (CI) and *p* values

Submodel	Predictor	Parameter	Polish data			UHZ data		
			Point estimate	95% CI	*p* value	Point estimate	95% CI	*p* value
Mean submodel	Intercept	*β*_0_	−1.428	(−1.890 to −0.965)	0.000	−1.176	(−1.269 to −1.083)	0.000
	Age	*β*_1_	0.034	(0.023 to 0.045)	0.000	0.013	(0.011 to 0.015)	0.000
	Sex	*β*_2_	2.169	(1.558 to 2.780)	0.000	0.851	(0.799 to 0.903)	0.000
	Age and sex interaction	*β*_3_	−0.029	(−0.044 to −0.014)	0.000	–		–
Precision submodel	Intercept	*γ*_0_	2.696	(1.937 to 3.454)	0.000	1.432	(1.394 to 1.470)	0.000
	Age	*γ*_1_	−0.030	(−0.046 to −0.014)	0.000	–		–
	Sex	*γ*_2_	−1.382	(−2.315 to −0.449)	0.004	–		–
	Age and sex interaction	*γ*_3_	0.032	(0.011 to 0.053)	0.003	–		–

Presented data describe model with age and sex as predictors which were further used for the simulation study

β, γ are regression parameters (please see the text for further details)

In the case of Polish data, the estimates of sex effect and age slopes were statistically significant. Also, interaction terms were statistically significant, meaning that age slopes are inferred to be different between males and females. The coefficient of pseudo-determination *R* ^2^ = 0.19. Figure 1a shows the interaction between age and sex in both mean and precision. In males, one can notice a very weak positive effect of age on the LVPWd and a weak negative age effect on dispersion of the LVPWd. In females, the effect of age on the LVPWd is much stronger and there is a positive age effect on dispersion of the LVPWd. The male median LVPWd increases from 9.67 to 9.82 mm, the female median LVPWd increases from 7.5 to about 8.9 mm, when age is varied from 20 to 50.

In the case of UHZ data, sex and age effect estimates on the LVPWd were statistically significant. The differences between males and females were present on the overall LVPWd level (Fig. 1b). In comparison to the Polish model, interaction terms between age and sex were not statistically significant; also there was no evidence that the dispersion of LVPWd is age- or sex-dependent. Consequently, the structure of the UHZ model is much simpler than that of its Polish counterpart. The coefficient of pseudo-determination *R* ^2^ = 0.19. The male median LVPWd increases from 8.4 to 9.0 mm, the female median LVPWd increases from 7.3 to about 7.8 mm, when age is varied from 20 to 50.

**Fig. 2:**
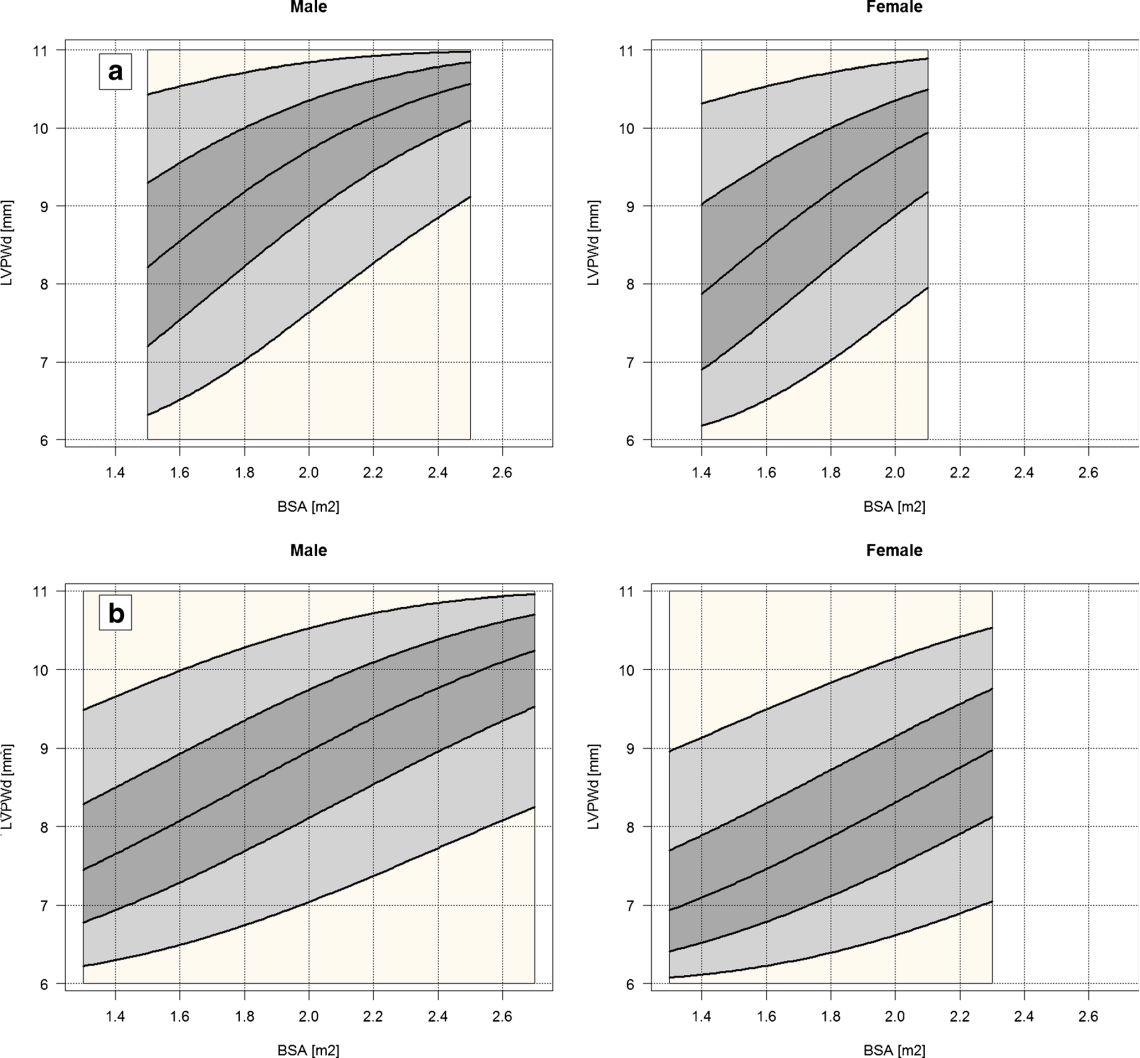
Relationship between LVPWd and BSA calculated with use of the developed models. **a** Polish data, **b** UHZ data. The *bold curves* from the *bottom* to *top* describe 5th, 25th, 50th, 75th and 95th conditional percentiles of the LVPWd, respectively

Estimation results of the second model are reported in Table 4. In this model, only BSA and sex were allowed to enter model equations, age was excluded. Figure 2 shows the estimated relationship between the LVPWd and both BSA and sex, separately for Polish and UHZ datasets. In the case of Polish data, it was found that in the presence of BSA, sex and interaction between sex and BSA were not significant, in both mean and precision equations, meaning that the estimated relationship between BSA and LVPWd is the same in males and females. The coefficient of pseudo-determination *R* ^2^ = 0.17, which is noticeably smaller than in the first model. Figure 2a shows a strong positive effect of BSA on LVPWd and a strong negative BSA effect on the dispersion of the LVPWd. Median LVPWd increases nonlinearly from 8.2 to about 10.1 mm for BSA increasing from 1.5 to 2.2 m^2^.

**Table 4:** Parameter estimates, 95% confidence intervals (CI) and *p* values — model with BSA and sex as predictors

Submodel	Predictor	Parameter	Polish data			UHZ data		
			Point estimate	95% CI	*p* value	Point estimate	95% CI	*p* value
Mean submodel	Intercept	*β*_0_	−3.372	(−4.332 to −2.413)	0.000	−3.201	(−3.480 to −2.923)	0.000
	BSAβ_1_	β_1_	2.128	(1.619 to 2.638)	0.000	1.534	(1.371 to 1.697)	0.000
	Sex	β_2_	–		–	0.452	(0.387 to 0.517)	0.000
	BSA and sex interaction	β_3_	–		–	–		–
Precision submodel	Interceptγ_0_	γ_0_	−0.218	(−1.481 to 1.046)	0.736	1.469	(1.431 to 1.507)	0.000
	BSA	γ_1_	0.824	(0.147 to 1.500)	0.017	–		–
	Sex	γ_2_	–		–	–		–
	BSA and sex interaction	γ_3_	–		–	–		–

β, γ are regression parameters (please see the text for further details)

In the case of the UHZ data, the mean submodel includes both BSA and sex, but not the interaction between them. There was no evidence that the dispersion of LVPWd is BSA- or sex-dependent. The coefficient of pseudo-determination *R* ^2^ = 0.22, which is higher than in the first model. Figure 2b shows a strong positive effect of BSA on LVPWd and level differences between males and females. The male median LVPWd increases from 7.9 to 9.4 mm, the female median LVPWd increases from 7.3 to about 8.8 mm, when BSA is varied from 1.5 to 2.2.

### Simulation Results

**Fig. 3:**
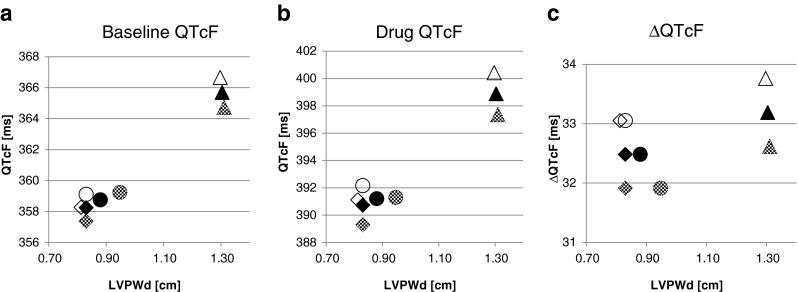
Simulated QTcF (**a** baseline, **b** with drug) and ΔQTcF values for three tested scenarios. *Diamond*, *circle* and *triangle* represent three tested scenarios — average value, current model predictions, and Sjögren model predictions of the LVPWd, respectively (*empty*, *shaded* and *filled symbols* represent women, men and all individuals, respectively)

Simulation results are presented in Table 5 and depicted in Fig. 3, separately for three tested scenarios. In the case of QT measurements, either with or without the presence of the tested drug, it can be noted that Scenario 2 (Sjögren model) resulted in a higher average QT as compared to the other two scenarios. Also, the use of the LVPWd models (Scenarios 2 and 3) resulted in a larger variability of QT as compared to the constant value scenario. However, the differences in variability between scenarios were almost nullified when the QT was corrected by the heart rate with the Fridericia equation. The differences in average QT levels between scenarios did not change after correction.

**Table 5:** Results of the BDMM-based simulations with use of the CSS system for various string length values (constant, Sjögren model derived and newly developed ASLPM model derived)

Simulation end-point		Scenario 1 (constant value)			Scenario 2 (Sjögren model)			Scenario 3 (ASLPM model)		
		All	Female	Male	All	Female	Male	All	Female	Male
String of cells length (mm)	mean	8.3	8.3	8.3	13.0	13.0	13.1	8.8	8.1	9.5
	SD	0	0	0	2.0	1.8	2.1	1.3	1.1	1.2
QT baseline — no drug (ms)	mean	345.7	344.4	346.9	352.8	351.6	354.0	346.1	343.6	348.7
	SD	3.7	2.0	4.5	4.2	3.2	4.8	4.8	3.2	4.8
QT — with drug (ms)	mean	377.0	376.1	377.8	384.8	384.0	385.6	377.4	375.1	379.8
	SD	2.8	1.9	3.2	3.8	3.5	3.9	4.2	3.2	3.7
QTcF baseline — no drug (ms)	mean	358.3	359.1	357.4	365.7	366.7	364.7	358.8	358.3	359.2
	SD	15.7	15.8	15.5	16.7	17.0	16.2	16.2	16.7	15.7
QTcF — with drug (ms)	mean	390.7	392.2	389.3	398.9	400.4	397.4	391.2	391.1	391.3
	SD	17.4	17.6	17.1	18.6	19.0	18.0	18.0	18.6	17.4
ΔQTcF (ms)	mean	32.5	33.1	31.9	33.2	33.8	32.6	32.5	33.1	31.9
	SD	2.6	2.6	2.4	2.8	2.8	2.6	2.6	2.6	2.4

## Discussion

The current study was preceded by an introductory data search study. The available scientific literature databases (Medline, Google Scholar, Scopus) were queried for publications describing the LVPWd in healthy individuals that were neither professional nor recreational athletes. The individuals were to be free of pathological cardiovascular changes, not carrying congenital heart pathologies and not taking any drugs related to cardiovascular disorders. Whenever possible, in addition to the LVPWd data, the age, gender, height, weight, BSA and BMI were collected. In total, 132 publications containing any relevant data were retrieved. The majority of publications was not primarily concerned with the LVPWd and consequently provided only aggregated data, i.e., mean and standard deviations, only a handful of publications provided a small number of individual data. After initial experimentation, it was found that the utilization of heterogeneous aggregate literature data to develop reliable LVPWd models poses a very challenging task. Therefore, it was decided to seek another source of information and perform modeling solely on individual data.

Two separate variable dispersion beta regression models (VDBRM) were developed, where age and gender as well as BSA and gender were shown to be statistically significant parameters describing the LVPWd. The LVPWd models were developed separately for the Polish and UHZ data sets. The main reason for this distinction was that we could envision a situation in which a cross-country comparison might be of interest to a reader as we had observed statistically significant differences between samples obtained from two countries (on average, 8.76 vs. 9.57 mm for males and 7.78 vs. 8.65 mm for females for Swiss and Polish data, respectively). Additionally, since the UHZ data set is more than 15 times larger than the Polish data set, it was clear that the models developed on the merged data set would be practically no different from the models developed on only the UHZ data set (results not presented), effectively hiding between-country differences. The modelling indicated that, in the case of Polish data, the male median LVPWd increases from 9.6 to 10.0 mm, and the female median LVPWd increases from 7.4 to about 9.3 mm, while in the case of UHZ data, the male median LVPWd increases from 8.4 to 9.0 mm, and the female median LVPWd increases from 7.3 to about 7.8 mm, in function of age (20–50 years). However, the cause of these phenomena is not clear and it is equally likely that it might be a result of differences in the studied population as well as being operator-dependent. Model including all three predictors namely age, gender, and BSA was also considered, however in terms of R^2^ it was no better than any of the bivariate models. The most important from the practical point of view is the model with age and gender as predictors since, for example, the distribution of both can be anticipated during the planning of a clinical trial thereby allowing the use of the BDM model in the clinical trials simulations. The BSA distribution among the clinical trial’s participants is less practical in use and possibly biased by the need of approximation. Consequently the models incorporating BSA (gender, BSA or gender, age, BSA) were deemed less relevant. However, as the relation between BSA and various measurement of heart structure is a frequent subject in the literature, such models were also presented.

As shown in this study, the models of the LVPWd can serve not only a descriptive purpose but can also be used to include inter-human differences in physiology into biophysically detailed mathematical models describing the electrophysiology of the human left ventricular heart wall. To test the sensitivity of the BDMM caused by the inclusion of the models of human LVPWd covering the physiological range of values, three competing approaches were investigated (Sjögren model and average LVPWd value against one of the newly established models). As it was presented in the 'Simulation results' section, the mean of ΔQTcF and its variability is comparable across all scenarios, leading to the conclusion that on the aggregate level, the inclusion of the LVPWd models does not change this important outcome. However, a closer look at the individual simulation results leads to a different conclusion, i.e., the inclusion of the models induces a positive correlation between the LVPWd and simulation outcomes, which brings them closer to reality by dismissing the previously made assumption of independence between LVPWd and electrophysiological outcomes. The LVPWd and the QTc correlation is close to linear; however, the ΔQTcF does not follow such a simple relation — this correlation is moderately nonlinear (result not shown). Differences between genders partially depend on the LVPWd model, although other BDMM’s parameters — e.g., main plasma ions concentrations, cardiomyocyte volume — also play a role. The physiological range of the QT is wide, spanning the range of 300–450 ms, dependent on age, gender, heart rate and other parameters [32–34]. Detailed analysis of such phenomenon would require a separate, thoroughly planned and conducted numerical experiment. Therefore, based on the simulated QT values it is not possible to particularly recommend any of the developed models as all of them meet the criteria specific for healthy individuals. There is, however, one element that should be taken into consideration when using biophysically detailed models of the cardiomyocytes for the drug safety assessment needs. Since they are planned to be used at an early stage of drug safety assessment as tools integrating in vitro studies results (drug-triggered ionic currents inhibition), their role is to mimic mainly healthy individuals [10]. In this case, currently proposed models would be recommended in opposition to the Sjögren model whose LVPWd predictions fall strongly above the current normal limits.

One of the limitations of the study is the assumption that the length of the cell string equals the heart wall thickness. In addition, the posterior location was assumed to be a place where the simulated electrophysiological phenomenon occurs. It is a clear oversimplification of the very complex physiological process. It is believed, however, to be adequate enough to be practically useful. Other limitation is connected with the unidimensionality (1D) of the employed BDMM. A 3D model of the heart, ideally with its geometry obtained with use of the MRI data, is necessary to compute physiological ECG, which in the current study was approximated by a surrogate, pseudo-ECG from 1D model [35, 36]. Such simplification was dictated mainly by the high computational cost of 3D approach which would render its practical large-scale application disputable.

## Conclusions

Physiological parameters influence the clinically observed drug-triggered ECG disruption and reactivity on drugs [25]. Therefore, the development of physiological parameters models, estimated with the use of rich, multicenter clinical data, can help to properly mimic populations involved in the clinical trials. Such new models, quantifying the relation between the LVPWd and the basic demographic and physiological parameters, were established and their performances were verified. Their usefulness was tested during the in silico study, where a model describing the left ventricular human cardiomyocyte electrophysiology was used for the electrophysiological endpoints simulation. As a result, the developed LVPWd models could be used in the in silico assessment of the drugs proarrhythmic potency assessment at the population level with the system data utilized.

## Electronic supplementary material

Below is the link to the electronic supplementary material.

ESM 1 (XLSX 478 kb)
